# Determinants of Plastic Waste Awareness, Preventive Practices, and Health Symptoms: An Urban‐Rural Comparative Cross‐Sectional Study in Bangladesh

**DOI:** 10.1002/hsr2.72363

**Published:** 2026-04-19

**Authors:** Md. Rownok Hasan, Abhijit Saha, Md. Sunyet Alam Chowdhury, Shovon Chakraborty, Irin Hossain

**Affiliations:** ^1^ Department of Occupational and Environmental Health (OEH) National Institute of Preventive and Social Medicine (NIPSOM) Mohakhali 1212 Dhaka Bangladesh

**Keywords:** attitudes, environmental exposure, environmental health, health knowledge, plastic waste, practice

## Abstract

**Background and Aims:**

Plastic waste is an escalating environmental and public health threat, particularly in Bangladesh, where rapid consumption and inadequate waste management exacerbate associated risks. Evidence on urban‐rural disparities in awareness, preventive practices, and related health outcomes remains limited. This study compared these differences and identified key determinants to inform context‐specific interventions.

**Methods:**

This comparative cross‐sectional study was conducted in 2022 among 768 respondents from urban (Dhaka) and rural (Tangail) settings. Households were selected using random route sampling, and eligible participants (≥ 18 years, both male and female, ≥ 1‐year residency) were selected through simple random sampling. Data were collected via face‐to‐face interviews using a pretested, semi‐structured questionnaire (Cronbach's *α* = 0.71–0.83). Primary outcomes were awareness, preventive practices, and self‐reported health symptoms within the past 12 months. Associations were examined using chi‐square tests, and determinants were assessed using multivariable logistic regression (AOR, 95% CI; *p* < 0.05).

**Results:**

Urban respondents demonstrated higher adequate awareness of environmental (79.4% vs. 59.1%) and health effects (80.7% vs. 45.3%; *p* < 0.001) of plastic waste. Preventive practices were also more common among urban residents (waste segregation: 44.8% vs. 18.6%; recycling practice: 34.7% vs. 11.1%). Self‐reported health symptoms comprised breathing difficulty (73.7% vs. 60.1%) and eye irritation (31.4% vs. 15.6%). In multivariable models, adjusting for age, sex, income, higher education, and urban residence was independently associated with greater engagement in recycling (AOR:3.13 and 3.57, respectively) and use of biodegradable alternatives (AOR:2.63 and 1.64, respectively; *p* < 0.001). They were also associated with a greater likelihood of avoiding polythene use driven by low cost or lack of alternatives (*p* < 0.05).

**Conclusion:**

Despite higher awareness among urban and educated populations, preventive practices remain limited, reflecting a persistent awareness‐practice gap shaped by structural and contextual constraints. Strengthening waste management infrastructure, regulating informal burning, and improving access to affordable alternatives are essential to mitigate plastic‐related environmental and health risks in Bangladesh.

## 1 Introduction

Plastic waste is an alarming global problem with serious environmental and health implications [1]. Although plastics provide substantial societal benefits, their leakage into the environment has become a major concern due to their persistence, ubiquity, and reliance on fossil‐derived feedstocks [2]. Global plastic production has increased steadily, reaching approximately 360 million tonnes annually, projecting continued growth over the coming decades [3]. Plastic pollution, first widely documented in aquatic systems in the 1970s, has since spread across terrestrial, freshwater, and marine ecosystems. If current trends continue, plastics may outweigh fish biomass in the oceans by mid‐century [4]. Single‐use plastics constitute a dominant source of coastal and marine litter, amplifying ecological risks in vulnerable habitats [5]. Social perceptions and awareness strongly influence consumption and disposal behaviors, and awareness‐raising interventions have been proposed as key mitigation strategies [6]. Mismanaged waste in densely populated coastal countries contributes disproportionately to ocean plastic inputs, with a limited number of nations accounting for most land‐based plastic entering the sea [5, 7, 8]. Addressing marine litter, therefore, requires coordinated technical, policy, and behavioral responses across global systems [8].

Plastic waste also contaminates soils, waterways, and food chains, prompting calls for improved waste management, product redesign, and behavioral change [9]. Effective mitigation depends on motivating consumer and community action, and evidence suggests that targeted communication strategies can increase engagement and reduce plastic pollution [10]. International and national initiatives underscore the urgency of action, including UN‐led efforts to substantially reduce plastic consumption and pollution [11]. In Bangladesh, multisectoral planning emphasizes sustainable plastic management through regulatory measures, infrastructure development, and public education [12].

Beyond environmental impacts, emerging evidence indicates that plastic pollution poses measurable risks to human health [13]. Humans are exposed to microplastics and plastic‐associated chemicals through ingestion, inhalation, and dermal contact [14, 15]. These exposures may result in short‐term (acute) health effects, including respiratory distress, coughing, eye irritation, skin irritation, and headache, particularly in settings where plastic waste is openly burned [15]. Open burning of plastic releases fine particulate matter (PM2.5 and PM10), dioxins, furans, black carbon, Polychlorinated Biphenyls, Volatile Organic Compounds (VOCs), Hydrogen Chloride, Hydrogen Cyanide, and Heavy Metals (Mercury), which can provoke airway inflammation and exacerbate pre‐existing respiratory conditions [15]. Long‐term (chronic) health risks are associated with sustained exposure to plastic‐derived pollutants and additives, including endocrine disruption, systemic inflammation, reproductive and developmental effects, and potential carcinogenicity [16, 17]. Chemical additives such as phthalates are endocrine‐active compounds that have been linked to metabolic and reproductive outcomes in occupational and environmental settings [16, 17].

Despite growing recognition of these exposure pathways, understanding the determinants of awareness, preventive practices, and health symptom reporting remains essential for designing interventions that reduce both plastic pollution and its associated health burden [11, 12]. In Bangladesh, urban and rural areas differ substantially in population density, waste generation patterns, access to formal waste collection, and availability of recycling infrastructure. Urban settings such as Dhaka experience high plastic consumption, informal recycling activities, and localized open burning. In contrast, rural districts such as Tangail often lack structured waste management systems and rely more heavily on household‐level disposal practices. However, empirical evidence directly comparing awareness, preventive behaviors, and exposure‐related health symptoms between urban and rural populations in Bangladesh remains limited. To address this gap, we conducted an urban‐rural comparative cross‐sectional study in Bangladesh. The study aimed to assess awareness, preventive practices, and self‐reported health symptoms related to plastic waste among urban and rural residents. Specifically, we sought to (1) evaluate awareness of plastic waste hazards; (2) assess preventive practices, including recycling and use of biodegradable alternatives; (3) compare urban‐rural differences in awareness, practices, and reported health symptoms; and (4) identify sociodemographic and exposure‐related determinants of preventive practices and reported health symptoms using multivariable logistic regression analysis. We hypothesized that urban residents would demonstrate higher awareness and better preventive practices, whereas exposure‐related health symptoms would vary according to environmental context and local waste management conditions.

## 2 Methods

### 2.1 Study Design

This comparative cross‐sectional study was conducted from 1st January to 31st December 2022 to assess urban‐rural differences in plastic waste awareness, preventive practices, and associated self‐reported health symptoms in Bangladesh.

### 2.2 Participants

Residents of Kamrangirchar‐Dhaka were classified as urban respondents, while residents of Tangail Delduar Upazila were classified as rural respondents. Individuals who had been residing in the study area for at least 1 year (The 1‑year residency criterion was applied to ensure sustained exposure to local waste‑management practices and environmental conditions, including plastic burning activities, that could influence chronic or subacute health symptoms as well as long‑term awareness and behavior), aged 18 years or older, both male female and willing to provide informed written consent were included and those who were severely ill or mentally unstable were excluded.

### 2.3 Variables

The questionnaire comprised 41 items across 8 domains: socio‐demographic variables (6 items), plastic waste environmental effects (6), plastic waste health effects (4), preventive and protective practices related to plastic exposure (7), self‐reported health symptoms of plastic waste exposure within the last 12 months (5), reasons for preferring plastic products (4), awareness of alternatives to plastic (4), and perceived strategies to reduce plastic waste (5). The questionnaire was pretested for clarity, relevance, and usability, and refined following participant feedback and expert review before finalization.

### 2.4 Data Sources/Measurement

Study places were purposively selected; Kamrangirchar in Dhaka district was chosen as the urban site, while Delduar Upazila in Tangail district represented the rural site. As the capital city area, Dhaka (Kamrangirchar) was selected to represent a densely populated urban setting characterized by high plastic consumption, informal recycling activities, localized open burning, and partial municipal waste management coverage. Tangail district was selected as a predominantly rural setting with lower population density, limited formal waste collection services, minimal recycling infrastructure, and greater reliance on household‐level disposal practices. These contrasting infrastructural characteristics were considered appropriate for examining urban‐rural differences in awareness, preventive practices, and exposure‐related health outcomes.

Within each site, the first household was selected using random route sampling to ensure unbiased initial selection. Starting points were randomly chosen from a list of enumeration areas. Interviewers followed a prespecified walking route and approached every second household. If no eligible respondent was present or if the household declined participation, the next household was approached. One eligible respondent per household was selected using simple random sampling according to the inclusion‐exclusion criteria. When multiple eligible individuals were present, a lottery method ensured random selection. Data were collected through face‐to‐face interviews using a pretested, semi‐structured questionnaire after obtaining informed written consent from all respondents. Cronbach's alpha indicated good internal consistency for environmental awareness (*α* = 0.83) and health awareness (*α* = 0.79), and acceptable reliability for preventive practice (*α* = 0.71), all exceeding the standard threshold of 0.70.

Health symptoms potentially related to plastic waste exposure were assessed using self‐reported responses. Participants were asked whether they had experienced symptoms such as breathing difficulty, eye irritation, skin irritation, headache, or nausea within the past 12 months. Respondents were further asked whether such symptoms occurred in settings characterized by visible plastic accumulation or open burning. The assessment was intended to capture perceived exposure‐related symptoms rather than clinically diagnosed conditions, and no medical verification was performed.

### 2.5 Bias

To minimize potential biases, a pilot study was conducted among 30 respondents from an area distinct from the study sites. This helped refine the questionnaire, reduce measurement error, and ensure a thorough pretest of the data collection process. Respondent anonymity was maintained to reduce social desirability bias. However, self‐reported health symptoms may have introduced recall and reporting bias, as symptoms were retrospectively reported and not clinically verified.

### 2.6 Study Size

A conservative prevalence of 50% (*p* = 0.5) was assumed to yield the maximum sample size and ensure adequate statistical power for estimating awareness and preventive practices in Bangladeshi settings.

The required sample size per group was calculated using the standard formula:

$$n=Z^{2}\times p\left(1-p\right)/d^{2},$$

where *n* is the required sample size per group, *Z* = 1.96 corresponds to a 95% confidence level, *p* = 0.5, and *d* = 0.05 represents the margin of error. This yielded a minimum sample size of 384 participants per group.

Accordingly, the total sample size was 768 respondents, with equal allocation to urban (*n* = 384) and rural (*n* = 384) groups.

### 2.7 Quantitative Variables

Socio‑demographic variables were assessed using open and closed‑ended questions. All awareness, practice, preference, and health‑symptom items were binary coded (No = 0; Yes = 1) to minimize respondent burden and reduce misclassification during face‑to‑face interviews, particularly among participants with lower literacy. Composite scores for environmental effects, health effects and preventive practices were constructed by summing the item responses for that domain and converting the raw score to a percentage; domain scores were then categorized using Bloom's cutoff criteria [18], with ≥ 80% classified as adequate/good, 60%–79% as moderate/neutral, and < 60% as inadequate/poor. The environmental effects domain comprised six items (maximum score = 6, minimum score = 0); so ≤ 60% = 3.6, 60%–79% = 3.6–4.74, and ≥ 80% = 4.8. For example, a raw score of five corresponds to 83.3% and is therefore classified as adequate (≥ 80%). Thresholds for the health effects and preventive practice domains were calculated similarly.

### 2.8 Statistical Analysis

Data were entered, cleaned, and analyzed using IBM SPSS Statistics version 25 (IBM Corp., Armonk, NY, USA). Statistical reporting followed the SAMPL guidelines and established principles for transparent reporting of observational research. All analyses were prespecified based on the study objectives, and no post hoc subgroup analyses were conducted. No missing data were observed in the final dataset; therefore, all analyses were performed on the complete sample.

Descriptive statistics were used to summarize the data. As the primary study variables were categorical, they were reported as frequencies and percentages, with corresponding numerators and denominators. Comparisons between urban and rural respondents were conducted using the Pearson chi‐square (*χ*²) test. For all inferential analyses, the test statistic and corresponding degrees of freedom were reported. All statistical tests were two‐sided, and a *p* < 0.05 was considered statistically significant.

Multivariable binary logistic regression analyses were performed to evaluate factors associated with selected binary outcome variables, including preference for polythene use due to lack of alternatives, preference for polythene use due to low cost, adequate awareness of microplastic formation, adequate awareness of plastic entry into the food chain, recycling practice, and use of biodegradable alternatives. Logistic regression modeling followed established methodological guidance [19]. Separate models were fitted for each outcome using maximum likelihood estimation.

Education level and place of residence were included as primary explanatory variables. Age (< 35/ ≥ 35 years), sex, and income were included as covariates based on conceptual relevance and bivariate associations. Adjusted odds ratios (AORs) with 95% confidence intervals (CIs) were reported to quantify effect sizes and precision.

Outcomes originally coded to reflect barrier conditions (e.g., preference for polythene products, lack of recycling, non‐use of biodegradable alternatives) were reciprocally transformed so that AORs represent positive behaviors and awareness levels for interpretative clarity. Given the cross‐sectional design of the study, as described in standard epidemiologic methodology [20], findings are interpreted as associative rather than causal.

### 2.9 Ethical Approval

The study was conducted in accordance with the ethical principles outlined in the Declaration of Helsinki and was approved by the Institutional Review Board (IRB) of National Institute of Preventive and Social Medicine (NIPSOM), Mohakhali, Dhaka‐1212, Bangladesh (IRB Approval Number: NIPSOM/IRB/2022/18/320). Informed verbal and written consent was obtained from all the study respondents.

## 3 Results

Table 1 demonstrates that urban and rural respondents differed significantly across most socio‐demographic characteristics. Rural participants were older overall, with 125/384 (32.6%) aged ≥ 48 years compared with 43/384 (11.2%) in urban areas (*χ*² = 29.576, df = 2, *p* < 0.001). Educational attainment showed marked disparity, with 167/384 (43.5%) of urban respondents having graduate‐level education compared with 17/384 (4.4%) of rural respondents (*χ*² = 143.956, df = 3, *p* < 0.001). Income and occupational distributions similarly differed (both *p* < 0.001). Gender distribution did not vary significantly between groups (221/384 vs. 208/384 male; *χ*² = 0.892, df = 1, *p* = 0.35). Family structure showed a modest difference, with nuclear families more common in urban areas (334/384 vs. 298/384; *χ*² = 4.796, df = 1, *p* = 0.03).

**Table 1:** Socio‐demographic characteristics of urban and rural respondents (*n* = 768).

Variable	Category	Urban (*n* = 384) frequency (%)	Rural (*n* = 384) frequency (%)	Test of significance *χ*²
Age (years)	18–32	246 (64.1)	140 (36.5)	*χ*² = 29.576, df = 2 ***p*** **< 0.001**
	33–47	95 (24.7)	119 (31.0)	
	≥ 48	43 (11.2)	125 (32.6)	
Gender	Male	221 (57.6)	208 (54.2)	*χ*² = 0.892, df = 1 *p* = 0.35
	Female	163 (42.4)	176 (45.8)	
Educational qualification	Illiterate/primary	21 (5.5)	205 (53.4)	*χ*² = 143.956, df = 3 ***p*** **< 0.001**
	Secondary	74 (19.3)	131 (34.1)	
	Higher secondary	110 (28.6)	31 (8.1)	
	Graduate and above	167 (43.5)	17 (4.4)	
Occupation	Unemployed	26 (6.8)	19 (5.0)	*χ*² = 84.902, df = 5 ***p*** **< 0.001**
	Housewife	62 (16.1)	103 (26.8)	
	Student	76 (19.8)	33 (8.6)	
	Service holder	133 (34.6)	14 (3.6)	
	Businessman	60 (15.6)	76 (19.8)	
	Day labor	26 (6.8)	138 (35.9)	
Monthly income (BDT)	< 10,000	53 (13.8)	281 (73.2)	*χ*² = 119.455, df = 2 ***p*** **< 0.001**
	10,001–30,000	234 (60.9)	91 (23.7)	
	≥ 30,001	97 (25.3)	12 (3.1)	
Family type	Nuclear	334 (87.0)	298 (77.6)	*χ*² = 4.796, df = 1 ***p*** = **0.03**
	Joint	50 (13.0)	86 (22.4)	

*Note:* Percentages are calculated within urban and rural groups (*n* = 384 each). All comparisons were two‐sided.

Abbreviations: *χ*², Chi‐square test; df, degrees of freedom.

Table 2 reveals that urban respondents consistently demonstrated higher awareness of the environmental consequences of plastic waste. Awareness that plastics enter oceans was reported by 331/384 (86.3%) urban respondents compared with 210/384 (54.7%) rural participants. Awareness of microplastic entry into the food chain showed the largest disparity (212/384 [55.2%] vs. 36/384 [9.4%]). Awareness of limited recycling capacity also differed markedly (305/384 [79.4%] vs. 129/384 [33.6%]). In contrast, awareness of burning‐related health risks was high in both groups (338/384 [88.0%] vs. 336/384 [87.5%]).

**Table 2:** Awareness of plastic waste consequences: Environmental and health impacts (*n* = 768).

Awareness variables	Urban (*n* = 384)		Rural (*n* = 384)	
	Yes (%)	No (%)	Yes (%)	No (%)
Environmental impacts				
Most plastics are single‐use	372 (96.9)	12 (3.1)	358 (93.2)	26 (6.8)
Plastic wastes enter oceans and waterways	331 (86.3)	53 (13.7)	210 (54.7)	174 (45.3)
Plastics break into microplastics and enter the food chain	212 (55.2)	172 (44.8)	36 (9.4)	348 (90.6)
Plastic bags clog sewers	351 (91.4)	33 (8.6)	265 (69.0)	119 (31.0)
Plastic waste promotes mosquito breeding and vector‐borne diseases	300 (78.1)	84 (21.9)	235 (61.2)	149 (38.8)
Recycling of plastics is limited	305 (79.4)	79 (20.6)	129 (33.6)	255 (66.4)
Health impacts				
Improper disposal increases disease risk	313 (81.5)	71 (18.5)	307 (79.9)	77 (20.1)
Chemicals from plastics affect the nervous and reproductive systems	244 (63.5)	140 (36.5)	143 (37.2)	241 (62.8)
Burning plastic causes respiratory illness and cancer	338 (88.0)	46 (12.0)	336 (87.5)	48 (12.5)
Plastic exposure may cause skin irritation	225 (58.6)	159 (41.4)	165 (43.0)	219 (57.0)

*Note:* Percentages are calculated within urban and rural groups (*n* = 384 each). All comparisons were two‐sided.

Table 3 shows that the preventive behaviors were more commonly reported in urban settings. Avoidance of polythene bags was reported by 109/384 (28.4%) urban respondents versus 73/384 (19.0%) rural respondents. Handwashing after plastic handling was practiced by 286/384 (74.5%) urban participants compared with 197/384 (51.3%) rural participants. Recycling behavior showed a threefold relative difference (133/384 [34.6%] vs. 43/384 [11.2%]). Community clean‐up participation was also higher in urban areas (202/384 [52.6%] vs. 128/384 [33.3%]).

**Table 3:** Preventive and protective practices related to plastic exposure (*n* = 768).

Preventive practices	Urban (*n* = 384)		Rural (*n* = 384)	
	Yes (%)	No (%)	Yes (%)	No (%)
Avoids polythene bags	109 (28.4)	275 (71.6)	73 (19.0)	311 (81.0)
Uses gloves or masks when handling plastic waste	151 (3)	233 (60.7)	70 (18.2)	314 (81.8)
Washes hands after handling plastic waste	286 (74.5)	98 (25.5)	197 (51.3)	187 (48.7)
Checks BPA‐free/food‐grade labels	158 (41.1)	226 (58.9)	68 (17.7)	316 (82.3)
Segregates household waste	172 (44.8)	212 (55.2)	71 (18.5)	313 (81.5)
Recycles plastic products	133 (34.6)	251 (65.4)	43 (11.2)	341 (88.8)
Participates in community clean‐up programs	202 (52.6)	182 (47.4)	128 (33.3)	256 (66.7)

*Note:* Percentages are calculated within urban and rural groups (*n* = 384 each). All comparisons were two‐sided.

In Table 4, substantial urban‐rural disparities were observed in composite awareness and preventive practice scores categorized using Bloom's criteria. Adequate environmental awareness was more frequent among urban respondents (305/384 [79.4%]) than rural respondents (227/384 [59.1%]; *χ*² = 39.205, df = 2, *p* < 0.001). Adequate health‐related awareness showed an even larger difference (310/384 [80.7%] vs. 174/384 [45.3%]; *χ*² = 111.456, df = 2, *p* < 0.001). Good preventive practice was reported by 63/384 (16.4%) urban respondents compared with 32/384 (8.3%) rural respondents (*χ*² = 12.633, df = 2, *p* = 0.002), although moderate practice predominated in both groups.

**Table 4:** Level of public awareness about environmental and health impacts of plastic waste and preventive practice (*n* = 768).

	Urban (*n* = 384) frequency (%)	Rural (*n* = 384) frequency (%)	Test of significance *χ*²
Level of awareness—Environmental effects of plastic waste; Score range (0–6)			
Adequate awareness (score ≥ 4.8)	305 (79.4)	227 (59.1)	*χ*² = 39.205 df = 2 ***p*** **< 0.001**
Neutral awareness (score 3.6–4.74)	58 (15.1)	102 (26.6)	
Inadequate awareness (score ≤ 3.6)	21 (5.5)	55 (14.3)	
Level of awareness—Health effects of plastic waste; score range (0–4)			
Adequate awareness (score ≥ 3.2)	310 (80.7)	174 (45.3)	*χ*² = 111.456 df = 2 ***p*** **< 0.001**
Neutral awareness (score 2.4–3.16)	48 (12.5)	138 (35.9)	
Inadequate awareness (score ≤ 2.4)	26 (6.8)	72 (18.8)	
Level of preventive practice; score range (0–7)			
Good Practice (≥ 5.6)	63 (16.4)	32 (8.3)	*χ*²=12.633 df = 2 ***p*** **=** **0.002**
Moderate Practice (4.2–5.53)	279 (72.7)	316 (82.3)	
Poor Practice (≤ 4.2)	42 (10.9)	36 (9.4)	

*Note:* Awareness and preventive practice levels were categorized using Bloom's cutoff criteria: ≥ 80% (adequate/good), 60%–79% (moderate/neutral), and < 60% (inadequate/poor) based on percentage scores.

Abbreviations: *χ*², Chi‐square test; df, degrees of freedom.

Table 5 presents that self‐reported symptoms associated with plastic exposure were more frequently reported in urban areas. Respiratory symptoms near burning plastics were reported by 283/384 (73.7%) urban respondents compared with 231/384 (60.2%) rural respondents. Sneezing or nasal irritation showed a similar pattern (251/384 [65.4%] vs. 160/384 [41.7%]). Differences were also observed for headache (143/384 [37.2%] vs. 88/384 [22.9%]) and eye irritation (121/384 [31.5%] vs. 60/384 [15.6%]).

**Table 5:** Self‐reported health symptoms to plastic waste exposure among urban and rural respondents (*n* = 768).

Health symptoms within the last 12 months	Urban (*n* = 384)		Rural (*n* = 384)	
	Yes (%)	No (%)	Yes (%)	No (%)
Breathing difficulty/cough near burning plastics	283 (73.7)	101 (26.3)	231 (60.2)	153 (39.8)
Headache/dizziness near burning plastics	143 (37.2)	241 (62.8)	88 (22.9)	296 (77.1)
Sneezing or nasal irritation near burning plastics	251 (65.4)	133 (34.6)	160 (41.7)	224 (58.3)
Eye irritation near plastic smoke	121 (31.5)	263 (68.5)	60 (15.6)	324 (84.4)
Skin irritation/rash after plastic contact	47 (12.2)	337 (87.8)	36 (9.4)	348 (90.6)

*Note:* Percentages are calculated within urban and rural groups (*n* = 384 each). All comparisons were two‐sided.

Table 6 presents perceptions regarding plastic use differed significantly by residence. Urban respondents more frequently cited availability (308/384 [80.1%] vs. 215/384 [55.9%]) and lack of alternatives (272/384 [70.8%] vs. 91/384 [23.6%]) as reasons for plastic preference (both *p* < 0.001), whereas lightweight characteristics did not differ (*p* = 0.82). Support for recycling initiatives was markedly higher in urban areas (319/384 [83.2%] vs. 143/384 [37.3%]; *p* < 0.001). In contrast, rural respondents more frequently supported imposing production penalties (281/384 [73.3%] vs. 246/384 [64.0%]; *p* = 0.006). Increasing plastic prices did not differ between groups (*p* = 0.65).

**Table 6:** Urban‐rural comparison of perceptions on plastic use, alternatives, and plastic waste reduction strategies (*n* = 768).

Variable		Urban (*n* = 384) frequency (%)		Rural (*n* = 384) Frequency (%)		*χ*²	df	*p*‐value
		Yes	No	Yes	No			
Reasons for preferring plastic	They are cheap	205 (53.4)	179 (46.6)	140 (36.6)	244 (63.4)	21.792	1	**<** **0.001**
	Easily available	308 (80.1)	76 (19.9)	215 (55.9)	169 (44.1)	51.588	1	**<** **0.001**
	Light weight	289 (75.2)	95 (24.8)	286 (74.5)	98 (25.5)	0.051	1	0.82
	Lack of alternatives	272 (70.8)	112 (29.2)	91 (23.6)	293 (76.4)	172.083	1	**<** **0.001**
Possible alternatives to Plastic	Jute/Cloth bags	348 (90.6)	36 (9.4)	358 (93.2)	26 (6.8)	1.658	1	0.20
	Paper	226 (59.0)	158 (41.0)	124 (32.3)	260 (67.7)	54.853	1	**<** **0.001**
	Glass	112 (29.2)	272 (70.8)	7 (1.9)	377 (98.1)	106.645	1	**<** **0.001**
	Biodegradable products	214 (55.7)	170 (44.3)	131 (34.4)	253 (65.6)	35.052	1	**< 0.001**
Strategies to reduce plastic waste	Banning plastic use	253 (65.8)	131 (34.2)	110 (28.6)	274 (71.4)	107.020	1	**< 0.001**
	Recycling	319 (83.2)	65 (16.8)	143 (37.3)	241 (62.7)	169.318	1	**< 0.001**
	Awareness campaigns	283 (73.9)	101 (26.1)	227 (59.0)	157 (41.0)	19.118	1	**<0.001**
	Increase the prices of plastics	158 (41.3)	226 (58.8)	165 (42.9)	219 (57.1)	0.206	1	0.65
	Impose a penalty for production	246 (64.0)	138 (36.0)	281 (73.3)	103 (26.7)	7.687	1	**0.006**

*Note: χ*² = Chi‐square test; df, degrees of freedom. Percentages are calculated within urban and rural groups (*n* = 384 each). All comparisons were two‐sided.

Table 7 presents the results of multivariable logistic regression analysis demonstrating that higher education and urban residence as consistent independent predictors of awareness and preventive behaviors. Higher education was strongly associated with adequate awareness of microplastic formation (AOR = 3.45, 95% CI: 2.44–5.00, *p* < 0.001) and engagement in recycling practices (AOR = 3.13, 95% CI: 2.22–4.35, *p* < 0.001). Urban residence independently predicted avoidance of polythene uses due to lack of alternatives (AOR = 4.17, 95% CI: 2.94–5.88, *p* < 0.001) and recycling engagement (AOR = 3.57, 95% CI: 2.50–5.00, *p* < 0.001). Higher income showed modest associations with avoidance behaviors and biodegradable product use. Age and sex were not independently associated with most outcomes.

**Table 7:** Determinants of plastic waste awareness, preferences and preventive practices (multivariable logistic regression analysis) (*n* = 768).

Outcome	Predictor	AOR	95% CI	*p*‐value
Avoidance of polythene use due to the lack of alternatives	Higher education	3.23	2.27–4.55	**<** **0.001**
	Urban residence	4.17	2.94–5.88	**< 0.001**
	Age ≥ 35 years	1.14	0.83–1.54	0.43
	Female sex	0.89	0.67–1.20	0.46
	Higher income	1.45	1.05–2.00	**0.02**
Avoidance of polythene use due to low cost	Higher education	1.59	1.16–2.17	**0.004**
	Urban residence	1.72	1.23–2.44	**0.002**
	Higher income	1.85	1.33–2.56	**< 0.001**
Adequate awareness of Microplastic formation	Higher education	3.45	2.44–5.00	**< 0.001**
	Urban residence	4.76	3.23–6.67	**< 0.001**
	Age ≥ 35 years	0.85	0.61–1.18	0.32
Adequate awareness of plastic entry into food chain	Higher education	1.96	1.43–2.70	**< 0.001**
	Urban residence	1.49	1.08–2.08	**0.02**
Engagement in recycling practice	Higher education	3.13	2.22–4.35	**< 0.001**
	Urban residence	3.57	2.50–5.00	**< 0.001**
Use of the biodegradable alternatives	Higher education	2.63	1.85–3.70	**< 0.001**
	Urban residence	1.64	1.18–2.27	**0.004**
	Higher income	1.39	1.02–1.89	**0.04**

*Note:* Separate multivariable binary logistic regression models were fitted for each dichotomous outcome. Education (0 = illiterate/primary, 1 = secondary or higher) and residence (0 = rural, 1 = urban). All models were adjusted for age (< 35/ ≥ 35 years), sex, education, residence, and household income. Reference categories were illiterate/primary education, rural residence, age < 35 years, male sex, and lower income. Outcomes are presented as positive behaviors/awareness levels; AORs were obtained via reciprocal transformation of the original barrier‐coded models. All *p*‐values are two‐sided, with statistical significance defined as *p* < 0.05.

Abbreviations: AOR, adjusted odds ratio; CI, confidence interval.

## 4 Discussion

This study provides comparative evidence on plastic waste awareness, preventive practices, and health‐related outcomes between urban and rural populations in Bangladesh. Although urban respondents demonstrated significantly higher awareness levels, preventive practices remained weak across both settings, indicating a persistent awareness‐practice gap. These findings underscore the importance of socio‐demographic and structural determinants in shaping plastic‐related behaviors and associated health risks, particularly in low‐ and middle‐income contexts.

### 4.1 Socio‐Demographic Determinants of Awareness and Preventive Practices

Urban residents demonstrated substantially higher environmental awareness (79.4%) than rural residents (59.1%) (*χ*² = 39.205, *p* < 0.001), while good preventive practices remained low in both groups (63/384 [16.4%] urban; 32/384 [8.3%] rural). Similar urban‐rural gradients have been reported in India, where higher awareness among urban households did not consistently translate into preventive behaviors [21]. In Bangladesh, Islam et al. likewise identified education and urban residence as significant predictors of plastic waste awareness [22]. Regression analyses further indicate that higher education and urban residence were independently associated with lower reliance on polythene products and greater engagement in preventive practices. Respondents with higher education and those residing in urban areas were more likely to avoid polythene products despite cost considerations or lack of alternatives. They were also more likely to engage in recycling and use biodegradable alternatives. Multivariable analyses demonstrated that higher education and urban residence independently predicted avoidance of polythene use, engagement in recycling, and adoption of biodegradable alternatives (Table 7), indicating that structural and informational advantages extend beyond descriptive urban‐rural differences observed in Table 4.

Consistent with these findings, studies from Turkey and other low‐ and middle‐income countries have reported strong associations between higher education and pro‐environmental awareness, alongside persistent constraints on behavioral adoption due to infrastructural and economic barriers [23, 24]. In Dhaka City, Rahman et al. similarly observed that despite higher awareness, preventive behaviors remained inconsistent because of service limitations [25]. Together, these findings indicate that education enhances awareness, while sustained behavior change depends on enabling structural conditions.

### 4.2 Awareness of Environmental and Health Impacts of Plastic Waste

Awareness of visible environmental impacts was high in both settings. Drain blockage due to plastic waste was recognized by (351/384, 91.4%) of urban respondents and (265/384, 69%) of rural respondents. Mosquito breeding was acknowledged by (300/384, 78.1%) of urban respondents. Comparable findings have been reported in Greece, where Latinopoulos et al. found strong public recognition of visible environmental consequences of plastic pollution [26]. However, awareness declined sharply for indirect and long‐term risks. Only (212/384, 55.2%) of urban and (36/384, 9.4%) of rural respondents recognized that plastics break down into microplastics that enter the food chain. The very low awareness of microplastics among rural respondents highlights a critical knowledge gap. Unlike visible plastic waste, microplastics represent an “invisible” environmental threat. Limited awareness of these systemic risks may contribute to higher rates of plastic burning and improper disposal in rural settings. Addressing this invisible‐risk perception gap should therefore be a priority in rural environmental education strategies. Similar knowledge gaps have been documented in Ethiopia, where Adane and Muleta reported limited understanding of downstream health effects despite high awareness of environmental nuisance [27]. Studies from Portugal and other European countries demonstrate higher awareness of indirect impacts, likely reflecting sustained public discourse and science communication [28]. The lower awareness of microplastics observed in this study may be attributed to limited public dissemination of emerging scientific evidence and the less tangible nature of chronic exposure pathways. Studies from Ethiopia and Ghana similarly report that communities primarily associate plastic waste with sanitation problems rather than systemic health risks [27, 29]. These findings highlight the need for targeted risk communication strategies that translate complex scientific concepts into accessible public messaging.

### 4.3 Awareness‐Practice Gap in Plastic Waste Management Behaviors

Despite higher awareness among urban residents, preventive behaviors remained suboptimal, reinforcing the awareness‐practice gap. This discrepancy may be explained by structural and contextual barriers beyond individual knowledge. In rural areas, limited access to formal recycling facilities, inadequate municipal waste collection services, the absence of segregated disposal systems, and limited market availability of affordable biodegradable alternatives may restrict behavioral change despite awareness. Conversely, in urban settings, high population density and environmental contamination may overwhelm individual preventive efforts. These findings suggest that awareness campaigns alone are insufficient without parallel improvements in infrastructure and policy enforcement. Global studies also indicate that awareness alone rarely leads to sustained behavior change in the absence of structural support. Thompson et al. emphasized that environmental concern often exceeds actual pro‐environmental behavior, particularly for plastic use [30]. In Malaysia, Chin et al. reported a similar gap, where over 80% of respondents acknowledged plastic pollution as a serious problem, yet fewer than 30% practiced consistent waste segregation [31]. Behavioral research from Australia suggests that convenience, habit, and perceived effort strongly mediate plastic‐related behaviors, even among environmentally aware populations [32]. Consumer studies from Hong Kong demonstrate that economic and convenience factors frequently outweigh environmental awareness in daily plastic use decisions [33].

### 4.4 Health Symptoms Associated With Plastic Waste Exposure

A notable proportion of respondents self‐reported health symptoms related to plastic waste exposure. Respiratory problems near burning plastic waste were reported by (283/384, 73.7%) of urban and (231/384, 60.2%) of rural respondents, while nasal irritation affected (251/384, 65.4%) of urban respondents. Urban respondents reported a higher proportion of symptoms, which may reflect differential exposure contexts and comparatively better practices than rural respondents. This pattern may reflect differential exposure contexts, such as proximity to informal waste burning sites, high population density, and clustered housing conditions in Kamrangirchar. In densely populated urban settings, limited air circulation may allow airborne pollutants from informal burning activities to accumulate, increasing ambient exposure levels. Consequently, individual preventive behaviors may be insufficient when community‐level environmental exposure remains substantial. International policy analyses have increasingly highlighted plastic pollution as an emerging public health concern rather than solely an environmental issue [31]. Rhein and Schmid reported that plastic additives and combustion by‐products may contribute to respiratory and systemic health effects, even in nonoccupational settings [32]. The World Health Organization has identified microplastics as a potential health risk, although causal evidence remains under development [33, 34, 35, 36].

### 4.5 Preference for Plastics and Adoption of Alternatives

Plastics were preferred primarily due to easy availability (308/384, 80.1% urban; 215/384, 55.9% rural) and low cost (205/384, 53.4% urban). Although awareness of jute and cloth bags exceeded 90% in both settings, knowledge of biodegradable alternatives was limited, particularly in rural areas (131/384, 34.4%). Similar affordability‐driven preferences have been documented in low‐income settings, where economic necessity constrains environmentally preferable choices [24, 27]. Evidence from high‐income countries suggests that adoption of alternatives increases substantially when cost‐competitive options are widely available and supported by policy incentives [32, 35]. This indicates that market and policy interventions are critical for reducing plastic dependence in Bangladesh.

### 4.6 Strategies for Plastic Waste Reduction and Policy Implications

Urban respondents favored recycling and awareness campaigns, whereas rural respondents showed stronger support for regulatory measures such as penalties on plastic production. Studies from Europe demonstrate that public acceptance of plastic reduction policies improves when interventions align with local priorities and infrastructure capacity [26]. Global frameworks emphasize that effective plastic waste reduction requires integrated strategies combining education, regulation, and waste management systems [34]. The observed urban‐rural differences further suggest that interventions should be tailored to local contexts. While urban areas may benefit from infrastructure strengthening and enforcement, rural settings may require regulatory measures and market incentives to reduce plastic availability. Aligning behavioral interventions with structural reforms will be crucial for achieving sustainable plastic waste reduction.

## 5 Conclusion

This cross‐sectional comparative study identified significant urban‐rural disparities in plastic waste awareness, preventive practices, and self‐reported health symptoms in Bangladesh. Although urban residents demonstrated higher awareness and greater engagement in preventive practice. However, they reported more exposure‐related symptoms, suggesting that environmental intensity may outweigh individual practices in densely populated settings. In contrast, rural populations exhibited lower awareness of microplastic‐related risks and faced substantial structural barriers, including limited access to formal waste collection, recycling facilities, and affordable biodegradable alternatives. These findings highlight a persistent awareness‐practice gap shaped not only by individual knowledge but also by infrastructural and market constraints. Strengthening municipal waste systems and regulating informal burning are critical priorities in urban areas, whereas rural strategies should focus on expanding service coverage, improving market access to affordable alternatives, and reinforcing regulatory oversight. Context‐specific, multi‐sectoral approaches that integrate infrastructure development, enforcement, and targeted risk communication are essential to reduce plastic‐related environmental and health risks.

## 6 Limitations

The purposive selection of study sites may limit the generalizability of findings to other regions of Bangladesh. Health outcomes were based on self‐reported symptoms and were not clinically verified, which may introduce recall, reporting, and misclassification bias. In addition, no objective environmental measurements or biomarker assessments were conducted to quantify plastic‐related exposures. Health symptoms were descriptively analyzed and not modeled using multivariable regression; therefore, potential confounding factors such as smoking, occupational exposures, indoor air pollution, and preexisting health conditions were not controlled for. Despite these limitations, the large comparative sample and consistency with prior literature strengthen the credibility of the findings.

## Author Contributions

**Md. Rownok Hasan:** conceptualization, methodology, software, data curation, investigation, validation, formal analysis, project administration, writing – original draft, writing – review and editing, supervision. **Abhijit Saha:** methodology, data curation, writing – review and editing, conceptualization. **Md. Sunyet Alam Chowdhury:** data curation, project administration, writing – review and editing, validation. **Shovon Chakraborty:** data curation, formal analysis, writing – review and editing, software, investigation. **Irin Hossain:** conceptualization, methodology, supervision, writing – review and editing, validation, formal analysis, project administration.

## Funding

The authors have nothing to report.

## Conflicts of Interest

The authors have no conflicts of interest to declare. All co‐authors have seen and agree with the contents of the manuscript. We certify that the submission is original work and is not under review at any other publication.

## Transparency Statement

The lead author Md. Rownok Hasan affirms that this manuscript is an honest, accurate, and transparent account of the study being reported; that no important aspects of the study have been omitted; and that any discrepancies from the study as planned (and, if relevant, registered) have been explained.

## Data Availability

The data that support the findings of this study are available from the corresponding author upon reasonable request. All authors have read and approved the final version of the manuscript. Md. Rownok Hasan had full access to all of the data in this study and takes complete responsibility for the integrity of the data and the accuracy of the data analysis.
